# TME18/486: Multimedia Pattern Sets in Telemedicine Systems

**DOI:** 10.2196/jmir.1.suppl1.e125

**Published:** 1999-09-19

**Authors:** E Kacki, J Stempczyñska

**Affiliations:** ^1^Technical University and Medical UniversityLódzPoland

**Keywords:** Telemedicine, Multimedia

## Abstract

The presented paper deal with two projects of telemedicine systems: 1) TELEONC system for oncology education; 2) postgraduated study health telematic system. The system projects have been created in close co-operation between the Institute of Computer Science at Technical University and the Department of Oncology at the Medical University of Lodz. The aim of the software of the systems is to improve efficiency and quality of education in medical study by creating broad acces to medical knowledge conveyed in a clear, easy-to-aquire way with the help of multimedia patern sets. The paper, after short description of the systems, has been devoted mainly to organization of sonic and image modules as well as their applications to problems of diagnosis and of drug informatons. The exploitation of multimedia in medical computer systems is extremely wide, from making use of natural voice during history-taking, through introduction of colour moving graphics illustrating main examinations, to the display of colour pictures of histopathological and cytological specimens. Apart from their written form, all results of auxiliary examinations, no matter whether of laboratory or picture type, can be given the shape of a drawing, moving chart or another attractive graphic form and displayed on the computer screen. Similarly, sound information obtaining from heart and lung auscultation can be introduced to the program. The most significant diagnostic examination in the oncological clinic is the microscopic one - cytological or histopathological. The discussion presented in the paper gives an idea how efficiently the communication with the systems assisting mcal diagnoses can be developed owing to the use of multimedia.

